# Supplementary material for: Rapid and Portable Detection of Hg and Cd in Grain Samples Based on Novel Catalytic Pyrolysis Composite Trap Coupled with Miniature Atomic Absorption Spectrometry
Source: Foods. 2023 Apr 25;12(9):1778. doi: 10.3390/foods12091778 (PMC10178322; doi:10.3390/foods12091778)
Supplement: Supplementary file 1 [file foods-12-01778-s001.zip › foods-2335030-supplementary.pdf]

Rapid and portable detection of Hg and Cd in grain samples based on novel catalytic pyrolysis composite trap coupled with miniature atomic absorption spectrometry

Tengpeng Liu [1], Jixin Liu [2,*], Xuefei Mao [1,*], Xiaoming Jiang [3], Yabo Zhao [2] and Yongzhong Qian [1]

[1] Institute of Quality Standard and Testing Technology for Agro-products, Chinese Academy of Agricultural Sciences, and Key Laboratory of Agro-food Safety and Quality, Ministry of Agriculture and Rural Affairs, Beijing 100081, China

[2] Beijing Ability Technology Co., Ltd., Beijing 100081, China

[3] Analytical & Testing Center, Sichuan University, Chengdu 610064, China

* Correspondence: ljx2117@gmail.com (J.L.); maoxuefei@caas.cn (X.M.)

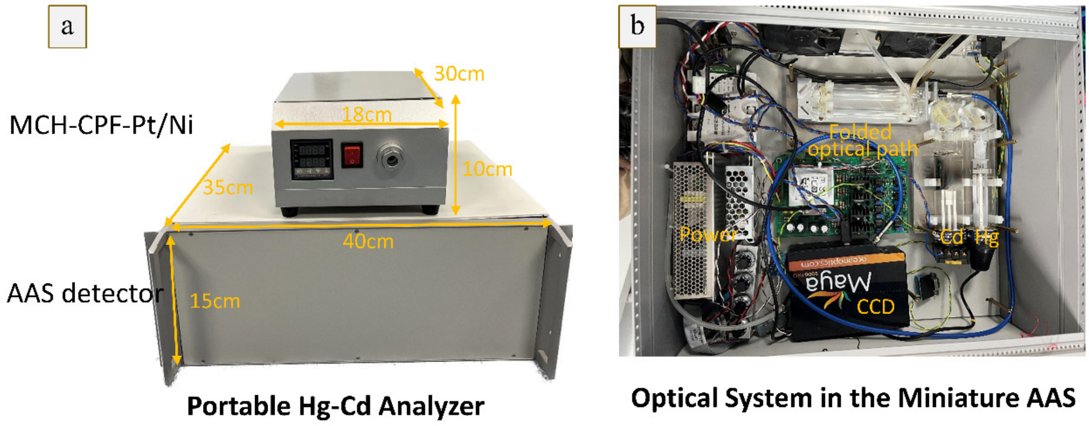

Figure S1. The picture of the portable Hg-Cd analyzer and the optical system in miniature AAS. **(Panel a)**: the portable Hg–Cd analyzer. **(Panel b)**: the miniature AAS. The whole portable Hg-Cd analyzer occupies approximately 14 kg in which the solid sampling module occupies 5 kg, and the AAS module occupies 9 kg; and 270 W are integrated for the total power, in which 30-100 W for MCH-ETV, 100 W for CPF, 140 W for *Pt/Ni* trap, and 30 W for AAS.

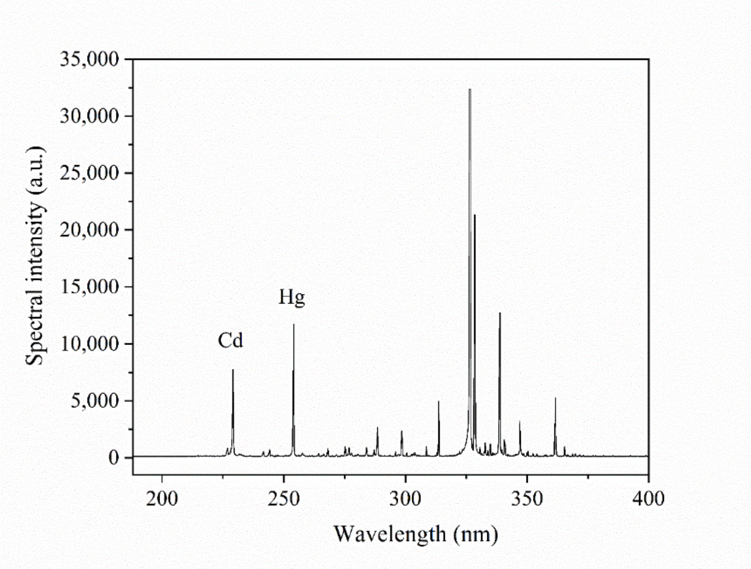

Figure S2. The background spectrum intensity of the miniaturized AAS. CCD integration time, 200 ms.

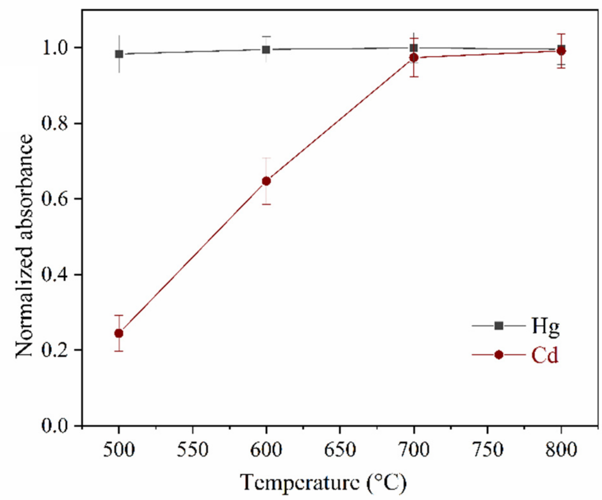

Figure S3. Effect of CPF temperature on Hg and Cd signals by MCH-CPF-*Pt/Ni*-AAS (n=3). A 10 μL Hg and Cd mixed standard solution (500 μg L^{-1}) was introduced, when without the CPF, the Hg and Cd signals were set as 1, and the other signals of Hg and Cd are normalized, respectively.

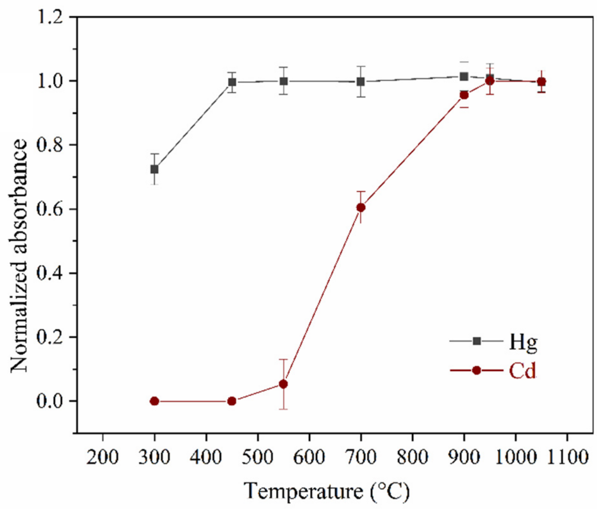

Figure S4. Vaporization of Hg and Cd in grain samples by the MCH-CPF-*Pt/Ni*-AAS (n=3). Herein, a 10 mg rice sample (R-03) was introduced into the MCH-CPF-*Pt/Ni*-AAS. The Hg and Cd absorbance are normalized with the signals at 450 °C (30 W) and 950 °C (100 W) set as 1, respectively.

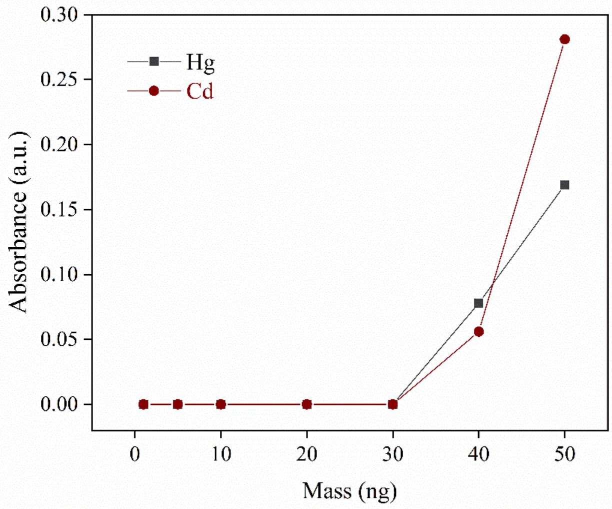

Figure S5. Trapping capacity of Hg and Cd using the *Pt/Ni* wafers. A series of mixed Hg and Cd standard solutions ranging from 0 to 5 mg/L (10 μL) were introduced into MCH-CPF-*Pt/Ni*-AAS, and the Hg and Cd breakthrough signals with *Pt/Ni* trap were measured.

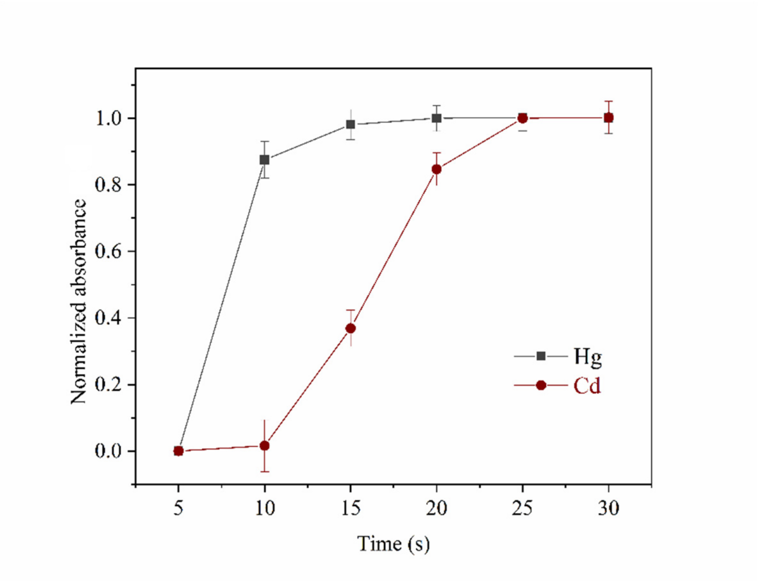

Figure S6. The effect of releasing time on Hg and Cd of the *Pt/Ni* trap. A 10 mg R-03 rice sample were introduced into the MCH-CPF-*Pt/Ni*-AAS, and released at 140 W under different *Pt/Ni* trap releasing times (5-30 s). The Hg and Cd absorbances were normalized with signal at 15 s and 25 s set as 1, respectively.

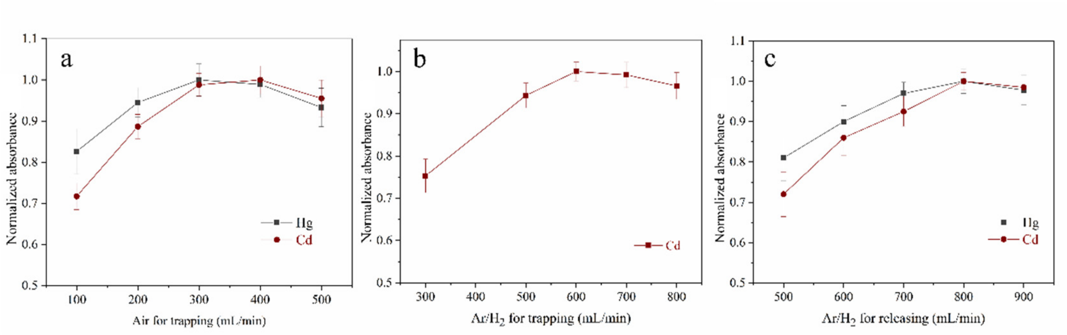

Figure S7. The effect of working gases on Hg and Cd detection (n = 3). Herein, a 10 mg R-03 rice sample was introduced into the MCH-CPF-*Pt/Ni*-AAS, the black and red lines are for Hg and Cd, respectively. **(Panel a)**: The air gas for Hg trapping and rice ashing, and the Hg and Cd intensities are both normalized with the signals at 300 mL/min set as 1, respectively. **(Panel b)**: The Ar/H_2 gas for Cd trapping, and Cd intensities are both normalized with the signals at 600 mL/min set as 1, respectively. **(Panel c)**: The Ar/H_2 gas for Hg and Cd releasing, the Hg and Cd intensities are both normalized with the signals at 800 mL/min set as 1, respectively.

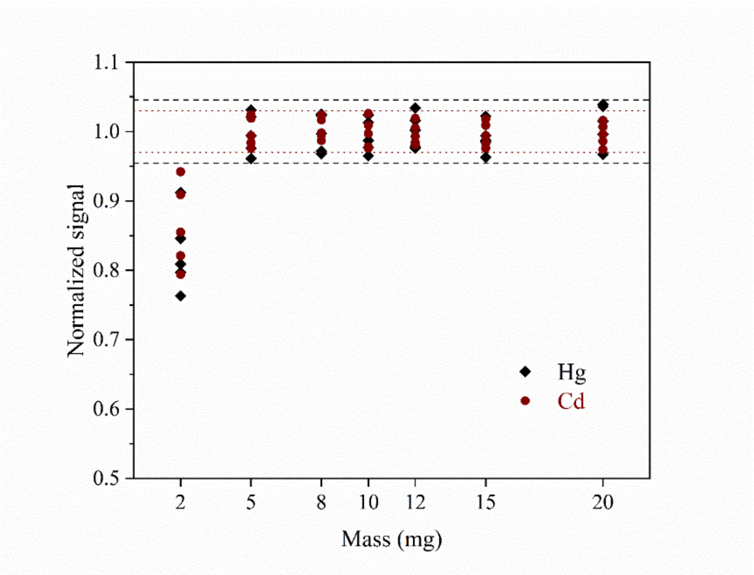

Figure S8. The evaluation of grain sample size using the proposed method (n = 5). The X-axis is sample mass, Y-axis is the normalized signal of Hg and Cd; and Hg and Cd certified values are normalized with different sample mass (2-20 mg) set as 1. The black and red broken lines are the lower and upper limits in the uncertainty ranges of Hg and Cd, respectively.

Table S1. Instrumental conditions of the Hg analyzer.

Programs	Temperature (°C)	Hold time (s)	O_2 flow rate (mL/min）
Drying	350	1	220
Ashing/trapping	800	70	220
Releasing	800	45	230
Cooling	0	10	230

Table S2. Instrumental conditions of microwave digestion ICPMS analysis.

**ICPMS**	**Parameters**
Incident RF power (W)	1500
Sampling depth (mm)	8
Scanning mode	Peak hopping
Cooling Ar flow rate (L/min)	15
Nebulizer Ar flow rate (L/min)	0.8
Auxiliary Ar flow rate (L/min)	0.4
Peristaltic pump (rpm)	30
Dwell time (ms)	30
Internal standard isotopes	^{115}In, ^{209}Bi
Isotopes of Hg and Cd	^{202}Hg, ^{111}Cd
CCT-KED	He
**Microwave digestion**	**Parameters**
80 °C	3 min
100 °C	3 min
130 °C	3 min
160 °C	3 min
180 °C	35 min
Cooling	30 min

To validate the method feasibility, 0.1 g grain samples were digested by HNO_3 (3 mL) - H_2O_2 (2 mL) mixture in PTFE vessels. The vessels were loaded into a microwave system for digestion as shown in Table S1, and then the digests were heated to 80°C to remove acid until <1 mL residue remained, subsequently accurately diluted to 25 mL with purified water for ICPMS detection.

Table S3. The interference of potential elements (n=3).

Interference materials	Added [a] (μg)	Recoveries (%)[b]	
		Hg	Cd
As	0.2	91	109
Pb	0.2	111	107
Zn	0.5	112	100
Cr	0.5	96	98
Mn	1	99	94
Ni	1	115	92
Fe	5	101	103
Cu	10	89	101
Mg	50	107	96
Ca	50	109	102
Al	50	104	107

[a] A series of rice sample (10 mg) containing interfering element (10 μL) with added levels mentioned above were measured by the proposed method.

[b] The recoveries are the ratio of measured Hg and Cd levels mixed with spiking elements by the proposed method to rice sample without spiking elements (n=3).
